# Pneumococcal Conjugate Vaccine impact assessment in Bangladesh

**DOI:** 10.12688/gatesopenres.12805.1

**Published:** 2018-04-26

**Authors:** Abdullah H. Baqui, Eric D. McCollum, Samir K. Saha, Arun K. Roy, Nabidul H. Chowdhury, Meagan Harrison, Abu Abdullah Mohammad Hanif, Nicole Simmons, Arif Mahmud, Nazma Begum, Salahuddin Ahmed, Ahad M. Khan, Zabed Bin Ahmed, Maksuda Islam, Dipak Mitra, Abdul Quaiyum, Miguel A. Chavez, Farhan Pervaiz, Catherine H. Miele, Holly B. Schuh, Rasheda Khanam, William Checkley, Lawrence H. Moulton, Mathuram Santosham

**Affiliations:** 1 Department of International Health, Bloomberg School of Public Health, Johns Hopkins University, Baltimore, MD, 21205, USA; 2 Department of Pediatrics, Eudowood Division of Pediatric Respiratory Sciences, School of Medicine, Johns Hopkins University, Baltimore, MD, 21205, USA; 3 Child Health Research Foundation, Dhaka, Bangladesh; 4 Johns Hopkins University, Bangladesh, Dhaka, Bangladesh; 5 North South University, Dhaka, Bangladesh; 6 International Centre For Diarrhoeal Disease Research, Dhaka, Bangladesh; 7 Division of Pulmonary and Critical Care, School of Medicine, Johns Hopkins University, Baltimore, MD, 21287, USA

**Keywords:** Pneumococcal conjugate vaccine, invasive pneumococcal disease, pneumonia, impact assessment, radiograph confirmed pneumonia, ultrasound confirmed pneumonia, Bangladesh

## Abstract

The study examines the impact of the introduction of 10-valent Pneumococcal Conjugate Vaccine (PCV10) into Bangladesh’s national vaccine program. PCV10 is administered to children under 1 year-old; the scheduled ages of administration are at 6, 10, and 18 weeks.

The study is conducted in ~770,000 population containing ~90,000 <5 children in Sylhet, Bangladesh and has five objectives: 1) To collect data on community-based pre-PCV incidence rates of invasive pneumococcal diseases (IPD) in 0-59 month-old children in Sylhet, Bangladesh; 2) To evaluate the effectiveness of PCV10 introduction on Vaccine Type (VT) IPD in 3-59 month-old children using an incident case-control study design. Secondary aims include measuring the effects of PCV10 introduction on all IPD in 3-59 month-old children using case-control study design, and quantifying the emergence of Non Vaccine Type IPD; 3) To evaluate the effectiveness of PCV10 introduction on chest radiograph-confirmed pneumonia in children 3-35 months old using incident case-control study design. We will estimate the incidence trend of clinical and radiologically-confirmed pneumonia in 3-35 month-old children in the study area before and after introduction of PCV10; 4) To determine the feasibility and utility of lung ultrasound for the diagnosis of pediatric pneumonia in a large sample of children in a resource-limited setting. We will also evaluate the effectiveness of PCV10 introduction on ultrasound-confirmed pneumonia in 3-35 month-old children using an incident case-control design and to examine the incidence trend of ultrasound-confirmed pneumonia in 3-35 month-old children in the study area before and after PCV10 introduction; and 5) To determine the direct and indirect effects of vaccination status on nasopharyngeal colonization on VT pneumococci among children with pneumonia **.  **

This paper presents the methodology. The study will allow us to conduct a comprehensive and robust assessment of the impact of national introduction of PCV10 on pneumococcal disease in Bangladesh.

## Introduction

Pneumonia is the leading cause of death among children 1–59 months of age globally ^1^. S *treptococcus pneumoniae* (pneumococcus) is a major cause of childhood pneumonia and other invasive pediatric diseases including meningitis and sepsis, accounting for approximately 14.5 million cases of invasive pneumococcal disease (IPD) and 826,000 child deaths worldwide ^2^. Among these estimated deaths, 741,000 are pneumonia deaths and 60,500 are deaths due to meningitis ^2^. Bangladesh is one of the ten countries with the highest number of IPD cases and IPD-related deaths among children under 5 years of age ^2^.

Pneumococcal conjugate vaccines (PCVs) have been documented to be safe and effective for reducing illness and deaths caused by *S. pneumoniae* in numerous studies. In most high-income countries, PCVs are used routinely, with a concomitant and substantial reduction of pneumococcal diseases ^3^,^5^. In 2006, the World Health Organization (WHO) recommended that PCV should be included in the routine infant immunization programs of all countries ^6^. Despite this recommendation, many low- and middle-income countries (LMICs) have not introduced the vaccine. The Global Alliance Vaccine Initiative (GAVI) co-financing has resulted in an increase in PCV introduction into national immunization programs of LMICs. The proportion of the world's birth cohort living in countries with PCV in national immunization programs has increased from 1% in 2000 to 31% in 2012 ^7^. One hundred twenty eight countries have included PCV in their national immunization programs, however global coverage for completing all three doses of the vaccine were only 37% in 2015 ^8^. This finding suggests that efforts to increase PCV introduction and use globally have made substantial progress; however, gaps in PCV use remain, particularly in Asia and in countries with large birth cohorts, where concerted efforts are required ^7^,^8^.

In 2013, the Ministry of Health and Family Welfare (MOHFW) of the Government of Bangladesh (GoB) decided to introduce 10-valent Pneumococcal Conjugate Vaccine (PCV10) into the national routine immunization program beginning in March 2015 making it the second South Asian country to implement PCV into routine childhood immunization, after Pakistan. In Bangladesh, the first dose of PCV10 is offered at 6 weeks of age along with pentavalent vaccine. The second and third doses are given at 10 and 18 weeks of age. Additionally, when the vaccine was introduced in March 2015, all infants who were less than 12 months old were offered the first dose of PCV10 and routine PCV10 immunization continued for this cohort. The decision to introduce PCV in Bangladesh in this way offered a unique and time-limited opportunity to generate pre-PCV data on pneumococcal diseases, and to conduct an impact assessment of PCV10 in the country following its introduction to measure its effect. Although Bangladesh had pre-PCV data on IPD from urban hospitals ^9^,^10^ allowing for before-after studies, population-based data were limited on culture-positive IPD from rural areas where the majority of child deaths are expected to occur ^11^.

We established community- and facility-based surveillance beginning January 1, 2014 to generate data on community-based pre-PCV IPD incidence rates in children 0–59 months of age in Sylhet district of Bangladesh. In addition to documenting the pre-PCV IPD rates in the study areas, the surveillance was designed to assess the feasibility of conducting an adequately powered IPD case-control study in our study population after vaccine introduction to estimate the impact of PCV10.

There are certain limitations of using IPD as the sole outcome when measuring the impact of PCV10. Many pneumococcal disease cases, particularly pneumonia cases, are not associated with bacteremia and therefore are not culture positive when testing for pneumococcus in the bloodstream ^12^,^14^. Thus, the etiology of community-acquired pneumonia can only rarely be determined by blood culture and relying on blood culture substantially underestimates pneumococcal disease burden. Widespread use of antibiotics in the community may further reduce the likelihood of positive results from blood culture. Although the point prevalence of clinical pneumonia among young children in Sylhet as determined by maternal report is fairly high at 4.9%, we expect to detect only about 50 IPD cases in our study area which contains about 90,000 <5 year old children in the year prior to PCV10 introduction ^15^. Therefore, it is important to consider other pneumococcal-related outcomes, albeit non-specific, when measuring the impact of PCV10 introduction. To provide a more complete assessment of the impact of the PCV10 vaccine, we are conducting a comprehensive impact assessment of PCV introduction in our population. We have designed this study to measure multiple outcomes including IPD, nasopharyngeal (NP) carriage, and both radiographically- and sonoraphically-confirmed pneumonia.

### Aims and objectives

The primary aim of this study is to conduct a comprehensive evaluation of the national introduction of the PCV10 in Bangladesh measuring multiple outcomes. To accomplish this aim, we have established the following objectives for the study:

**Objective 1:** To collect data on community-based pre-PCV incidence rates of IPD in children 0–59 months of age in Sylhet district of Bangladesh.

**Objective 2:** To evaluate the effectiveness of PCV10 introduction on Vaccine Type (VT) IPD in children 3–59 months of age using an incident case-control study design. Secondary aims include measuring the effects of PCV10 introduction on all IPD, regardless of serotype, in children 3–59 months of age using the case-control study design, and quantifying the emergence of Non Vaccine Type (NVT) IPD.

**Objective 3:** To evaluate the effectiveness of PCV10 introduction on chest radiograph-confirmed pneumonia in children 3–35 months of age using an incident case-control study design. An additional aim is to estimate the incidence trend of clinical and radiologically-confirmed pneumonia in 3–35 month-old children in the study area before and after introduction of the PCV10 ** vaccine.

**Objective 4:** To determine the feasibility and utility of lung ultrasound for the diagnosis of pediatric pneumonia in a large sample of children in a resource-limited setting. We also sought to evaluate the effectiveness of PCV10 introduction on ultrasound-confirmed pneumonia in children 3–35 months of age using an incident case-control study design and to examine the incidence trend of ultrasound-confirmed pneumonia in 3–35 month-old children in the study area before and after introduction of the PCV10 vaccine.

**Objective 5:** To determine the direct and indirect effects of vaccination status on NP colonization on VT pneumococci among children with pneumonia.

## Protocol

### Study design

This is a population-based prospective community- and facility-based observational study with a number of nested studies including incident case-control and incident trend studies to evaluate the impact of PCV10. To conduct the impact assessment, we are considering four outcomes: 1) IPD; 2) chest radiograph-confirmed pneumonia, 3) lung ultrasound-confirmed pneumonia; and, 4) NP carriage.

### Study site and population

**Figure 1.**
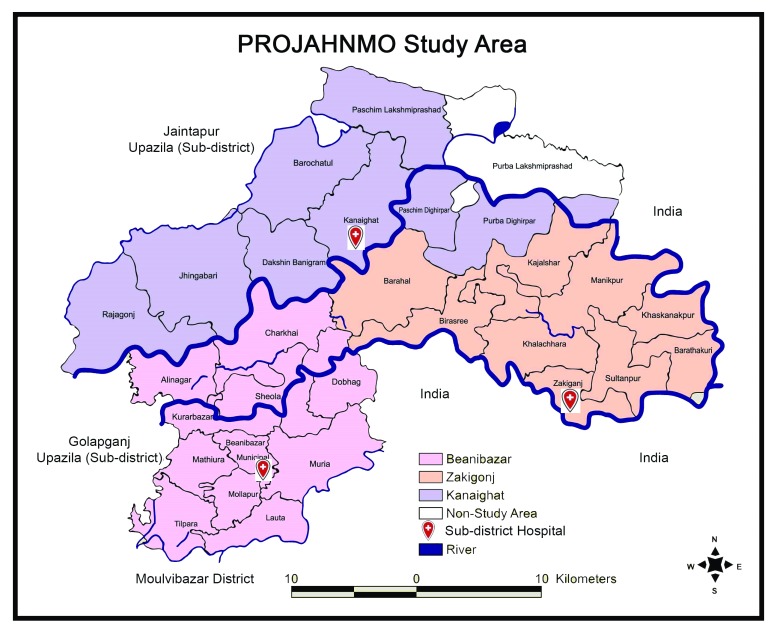
Study area. Pneumococcal Conjugate Vaccine impact assessment in Bangladesh.

The PCV Impact Assessment in Bangladesh study is being conducted in the Projahnmo study group’s research site in the Sylhet district of rural Bangladesh. The Projahnmo study group is a research partnership of the Johns Hopkins University with the Government of Bangladesh’s Ministry of Health and Family Welfare (MOHFW) and several Bangladeshi non-governmental organizations (NGOs): the International Centre for Diarrhoeal Disease Research, Bangladesh (iccdr,b); Shimantik; and Child Health Research Foundation (CHRF). The field site was established in 2001 to contribute to the improvement of maternal, newborn and child health by conducting clinical-epidemiological studies, intervention trials and program evaluations. The study area covers three *upazilas* (sub-districts) of Sylhet district of Bangladesh (Zakiganj, Kanaighat and Beanibazar) with an estimated population of 770,000 (data source: project census, 2011), yielding an annual birth cohort of approximately 19,725 ( Figure 1). All households and women of childbearing age in the study area have unique current and permanent identification numbers, which allow for individual tracking and longitudinal follow-up. The study is being conducted among children 0–59 months old in this active surveillance area.

The substantial existing infrastructure includes a census; a Global Positioning System-based map and background characteristics of the entire population; community- and facility-based surveillance; mechanisms for community-based sampling and case identification; referral, specimen collection, transport; state-of-the-art laboratories; and a data center. We have established community-based surveillance, which involves home visits by trained community health workers (CHWs) in the entire area in about 90,000 <5 year old children. Working closely with the Bangladesh MOHFW, we have established facility-based surveillance within the MOHFW sub-district hospital outpatient clinics and inpatient pediatric wards in the study area for potential IPD case detection, specimen collection, and transport. Project physicians who are trained and standardized to the study research protocols staff these facilities.

### Objective 1: Establish pre-PCV IPD rate

Trained female CHWs who are residents of the community and have at least a 10th grade education have been assigned to a population of about 10,000 individuals. Each CHW visits all households (about 2,000 households on average) in her area once every two months to update the population data by recording all new pregnancies, births, deaths, marriages and movements. Background data on age, parity, literacy, prior obstetric history and socio-economic status of all enrolled women in our study area were collected as part of previous or ongoing studies and are available in our database ^16^,^20^. These data are collected for all newly identified pregnant women who did not provide them previously. Similarly, dates of birth of children born in previous or ongoing studies are available in our database and the data are prospectively collected for women and children <5 years of age who were not part of prior studies. In our efforts to provide a standard package of care to pregnant women and newborns in this research site, CHWs provide information to pregnant women about care during pregnancy, delivery and the postpartum period during regular visits every two months. Families are encouraged to notify CHWs about all births as soon as possible using the existing cell phone-based birth notification system, and are encouraged to seek care from study designated hospitals for any illnesses in newborns and children. All children are visited every two months until they are 60 months of age, and data on their vital status, illness history and care seeking are recorded. Mothers and family members are educated during home visits every two months on signs of pneumococcal diseases (pneumonia, meningitis, and other IPD) and are further encouraged to visit the designated study hospitals for clinical evaluation, enrollment and management of suspected illnesses of the children.

The CHWs also recruit and supervise local resident village health workers (VHWs) in each village in the study area. The VHWs are provided with thermometers marked at a cutoff level of 101°F and trained on their use. They are also trained to recognize symptoms of pneumonia and meningitis. In addition, they visit and screen children of consented parents weekly for fever, respiratory problems and danger signs, and refer sick children to one of the study hospitals. The VHWs receive a monthly stipend and financial incentive payments for successful referrals of eligible children with clinical pneumonia, high fever, and meningitis. VHWs do not collect any study data but facilitate case detection and referral from surveillance population to the study hospitals. In case of refusal of referral advice by families, the VHWs call the CHWs for further facilitation of the referral process. The CHW referral criteria is in Table 1.

**Table 1.** Referral criteria for pneumonia and invasive pneumococcal diseases at the community level.

High fever (≥101.0°F)
Hypothermia (<95.9°F)
Movement only on stimulation
No movement/unconscious
Reported/Observed convulsions
Unable to feed
Vomits everything
Bulging fontanelle
Stiff neck
Lower chest indrawing
Observed head nodding/use of neck muscles in breathing
Noisy breathing
Cough/difficulty in breathing PLUS Fast breathing
H/O Scanty micturition or high colored urine PLUS Puffy face/Ascites/leg edema

In addition to family education for illness recognition and care seeking from sub-district hospitals, we have developed a network with the first-level facilities and private health care providers in both the formal and informal sectors within the study area and encourage them to refer sick children with suspected pneumococcal disease to the designated study hospitals. We have also included Sylhet Medical College and selected private tertiary level referral hospitals in Sylhet City in this network, which are frequent referral sites for children with symptoms of meningitis in our study area. We have mapped the first-level clinics and private health care providers in the study area including MOHFW’s Family Welfare Centers (FWCs), Community Clinics, NGO-supported clinics, village doctors and drug sellers. All of these providers are given an orientation on the study and are requested to refer eligible cases to the designated hospitals. The orientation includes standardized definitions of suspected pneumonia and meningitis cases and instruction on keeping a list of cases by village. Our study physicians periodically visit these providers and facilities to assess the quality of diagnoses made and reinforce adherence to the case definitions and protocols for referral of all suspected cases to designated health facilities. We identify community providers who see a particularly high volume of children <5 years of age with lower respiratory symptoms, and obtain the providers’ consent to station mobile teams of trained health workers at their offices. We have established a quality control program to ensure the quality of screening to maximize enrollment of eligible cases and of blood specimen collection to avoid specimen contamination. Periodic and need-based refresher trainings are organized for all study staff.

For referred cases of meningitis being treated in non-study hospitals, a trained study physician travels to visit the patient in that hospital, obtain parental informed consent, clinically screen the patient for suspected meningitis, and perform lumbar puncture if appropriate and if the parent provides consent. The cerebrospinal fluid (CSF) specimen is transported to the study laboratory using the procedures described below.

Upon presentation at any of the participating study hospitals, a research assistant requests parents/caregivers of all children <5 years of age to give consent for screening for suspected IPD (clinical pneumonia, meningitis, or high fever) and for collection of basic demographic and clinical information. Suspected IPD cases are identified by physicians at the study hospitals and mobile team members in the community. After obtaining written informed consent from parents, detailed data on demographic characteristics, clinical assessment, and history of medication use are recorded in a case report form (CRF). The criteria used for blood or CSF collection are provided in Table 2.

**Table 2.** Criteria for blood or CSF collection for testing for invasive pneumococcal diseases.

**Inclusion criteria**	1. Any of following danger signs i. Hypothermia (<95.9°F) ii. Movement only on stimulation iii. No movement/unconscious iv. Reported/Observed convulsions v. Unable to feed vi. Vomits everything vii. Bulging fontanelle viii. Stiff neck
	2. High fever (≥101.0°F)
	3. Moderate fever (99.5–100.9) PLUS Any of the following signs of severe pneumonia i. Chest indrawing ii. Observed head nodding during breathing iii. Stridor (persists after bronchodilatation)
	4. Suspected Nephrotic syndrome or Glomerulonephritis i. History of scanty micturation or high colored urine AND Oedema (Puffy face/Leg oedema/Ascities)
**Exclusion criteria**	1. Previously enrolled in the preceding 7 days
	2. Had received antibiotics doses (confirmed by prescription OR bottle/strip) and the last dose within last 24 hours

A blood sample (~3 ml) is collected from each eligible child and half of it is directly inoculated into a BACTEC (Becton Dickinson Diagnostic Instrument Systems, Sparks, MD, USA) pediatric blood culture bottle and kept at room temperature until transportation to the Sylhet laboratory, where it is processed in a BACTEC machine. The culture bottles that show signs of bacterial growth are then subcultured. The other half of the blood specimen is transported to the Sylhet laboratory maintaining cold chain for molecular testing.

Lumbar puncture is performed for all consented hospitalized cases with signs of meningitis. Up to 2 ml of CSF is collected for immediate plating on chocolate, blood and MacConkey agar media and a portion of CSF is saved for cytology and measurement of biochemical parameters. The remaining CSF specimen is preserved for molecular testing. All specimens are transported to the study laboratory in Sylhet at least twice daily for identification and real-time assessment of biological parameters. Preliminarily identified isolates are transported to the CHRF lab at Dhaka Shishu Hospital in Dhaka for reconfirmation, serotyping, and drug susceptibility testing. All molecular testing is performed in the CHRF lab in Dhaka using real-time polymerase chain reaction (PCR) techniques. The study doctors record data on treatment given, status at discharge and laboratory findings in the CRF. An IPD case is identified using a pre-determined criteria that take in to account the clinical findings, culture and PCR results. All sick children receive treatment based on the physicians’ clinical diagnosis. For outpatients, a written report on blood culture results is provided to parents at the next routine household visit after the results become available.

### Objective 2: IPD case-control and incident trend studies

We have maintained the IPD surveillance established for Objective 1 in our study area, including community surveillance, health facility activities and laboratory procedures, in order to detect IPD cases. The inclusion criteria for the case-control and incident trend studies are shown in Table 3 and Table 4. The age eligibility for enrollment in the IPD case-control study is shown in Table 5 and the sample size requirement for IPD case-conrol study is shown in Table 6.

**Table 3.** Inclusion criteria for the IPD case-control study.

**Inclusion criteria**	1. Age 3–59 months meeting age-eligibility criteria under which they have had the potential to have started the PCV vaccine (not born yet or under age 12 months on March 25, 2015).
	2. Resident of the active surveillance area clinically eligible. a. For cases – clinically suspected invasive pneumococcal disease (pneumonia or meningitis or high fever) and pneumococcus isolated form blood or cerebrospinal fluid b. For controls-without any signs/symptoms of invasive pneumococcal disease as screened by physicians (hospital controls) or data collectors in the community (community controls)
	3. Written informed consent by parents/care givers.
**Exclusion criteria**	1. Refusal to join the study

**Table 4.** Inclusion criteria for the incident-trend study.

**Inclusion criteria**	1. Age 3–59 months meeting age-eligibility criteria under which they have had the potential to have started the PCV vaccine (not born yet or under age 12 months on March 25, 2015).
	2. Resident of the active surveillance area
	3. Diagnosed clinically as invasive pneumococcal disease and pneumococcus isolated from blood or cerebrospinal fluid
	4. Written informed consent for participation in x-ray study by parents/care givers.
**Exclusion criteria**	1. Refusal to join the study

**Table 5.** Age-eligibility criteria for inclusion in invasive pneumococcal diseases case-control study by study months. Age in completed months.

Year	2015					2016											
Month	Aug	Sep	Oct	Nov	Dec	Jan	Feb	Mar	Apr	May	Jun	Jul	Aug	Sep	Oct	Nov	Dec
Month of case- control study	1	2	3	4	5	6	7	8	9	10	11	12	13	14	15	16	17
Minimum age of eligibility	3	3	3	3	3	3	3	3	3	3	3	3	3	3	3	3	3
Maximum age of eligibility	15	16	17	18	19	20	21	22	23	24	25	26	27	28	29	30	31
Year	2017																
Month	Jan	Feb	Mar	Apr	May	Jun	Jul	Aug	Sep	Oct*	Nov*	Dec*	*If needed for adequate sample *size*				
Month of case- control study	18	19	20	21	22	23	24	25	28	29	30	31					
Minimum age of eligibility	3	3	3	3	3	3	3	3	3	3	3	3					
Maximum age of eligibility	32	33	34	35	36	37	38	39	40	41	42	43					

**Table 6.** Sample size estimates for detectable levels of vaccine effectiveness in invasive pneumococcal diseases case-control study.

	90% Vaccine Effectiveness (VE)			95% VE		
	Average coverage	Number of VT IPD cases	Lower bound	Average coverage	Number of VT IPD cases	Lower bound
No vaccine	0%	45	N/A	0%	45	N/A
Scenario 1 (simulation)	45%	25	50.8	45%	24	72.7
Scenario 2	50%	25	54.3	50%	23	73.9
Scenario 3	60%	21	51.8	60%	19	72.1
Scenario 4	70%	17	44.2	70%	15	67.1

For each enrolled IPD case child, we randomly select and enroll two sets of four matched control children from the study area. The control children are of the same sex as the case and within ±1 month of age of the case child. The first set of controls is selected from the hospital from where the case is detected. Four age- and sex-matched children are selected from among those who visit the same health facility within 1 week of the identification of the case and are screened as not having any symptoms or signs of clinical pneumonia or meningitis. At our three sub-district hospitals, consent for screening of all children under 5 years of age is obtained at the time of presentation to the hospital. Children who screen negative for symptoms of suspected IPD are placed in a pool of possible controls. If a case is identified during a given week, the age- and sex-matched controls are then selected and visited at home to collect background data including immunization status data. For meningitis cases identified at tertiary hospitals in Sylhet, age-and sex-matched children are identified at the pediatric surgery clinic and at other services that are not treating children with infectious disease during the two weeks following identification of the case. Children of parents who consent for their children to serve as controls are recruited to the study.

 The second set of controls is selected from the community. Four controls matched to the case child by age, sex, and road distance from their home village to the study hospital where the case was ascertained are selected within one week of identification of the case. To facilitate random selection, we use the complete list of 823 villages (including municipal wards) in our study area, each of which has a population of about 1,000 people on average. All the villages are uniquely numbered. Once a case is identified, we generate a complete list of villages within the hospital catchment area that are within one kilometer of the distance from hospital to the village where the case is ascertained (the “index village”). If fewer than four matching villages are generated, we then expand the matching distance range to two kilometers. If we have more than four villages in the list, we randomly rank the villages and attempt to select one control per village using the ranked list. The children within each village who meet the age- and sex-matching criteria for the case are also listed and randomly ordered. The health worker then sequentially visits each child and asks the parent for their consent to screen the child as a control. The screening involves an examination for signs of IPD (pneumonia, high fever, and meningitis) and maternal recall of the presence of any such symptoms during the past seven days. Once one healthy control has been identified in a given village, we move to the second-ranked village to select the second control, and so forth.

Informed written consent is obtained from the parents of both hospital and community controls. After consent is obtained, the same information that we collect from a case child is collected from each of the control children. The information on the vaccination status of the control children are collected using the same method used for case children.

***Exposure ascertainment.*** The following epidemiologic information is collected for each case and control child using a standardized data collection instrument:

- Risk factors for IPD: age, family structure, antibiotic use, season/date, comorbid illness status of the individual, crowding measures, smoke exposure variables, geographic location, nutritional status, birth weight, gestational age at birth, and socio-economic status;
- Vaccination status: Immunization status of each child including PCV, oral polio vaccine, and pentavalent vaccine is recorded. The mother is asked to provide the child’s immunization card for examination and it is extracted onto a standard data collection form. We also take a digital photograph of the immunization card. If the immunization card is not available, the caregiver is asked if the child has been vaccinated, and if so, at which immunization center. The register of the identified immunization clinic is then reviewed and the child’s immunization history is abstracted. Only children who received at least two doses of the vaccine at least fourteen days before the date of case identification or control selection will be considered as vaccinated for the purposes of the study analysis;
- Anthropometric measurements: Current weight, height and mid-upper arm circumference are also collected.

### Objective 3: Chest radiograph confirmed pneumonia case-control and incidence trend studies

We have established pediatric computed radiology (CR) units in the three sub-district hospitals through procurement of portable analogue radiograph units (POLYMOBIL® Plus, Siemens, Erlangen, Germany) with accompanying CR Fujifilm™ cassette readers, which digitize the image. Trained radiography technologists have been recruited by the project and are responsible for carrying out supine antero-posterior chest radiographic imaging for children meeting the study clinical pneumonia criteria. Training on the use of the radiograph unit and CR cassette readers was conducted online via teleconference with the vendors. The radiographers have been provided with hands-on training by radiology faculty and technologists from Sylhet Medical College and Dhaka Shishu (pediatric) hospital. We have a service, inspection and maintenance agreement for the radiograph units with the vendor (Siemens/Bangladesh) to ensure that the radiograph machines are in good working order at all times and meet all safety requirements. The machines are calibrated and the performance of the machines are evaluated immediately following installation, immediately following any repair, parts replacement or maintenance, and annually. Radiography technicians are trained to capture radiograph images according to a standardized protocol and a random sample of images are assessed for quality weekly by the study pediatric pulmonologist (EDM).

Study physicians at the study hospitals are also trained to assess image quality. We aimed for a proportion of less than 5% “uninterpretable” images assessed by a panel of chest radiograph readers. For children 3–35 months of age who meet the inclusion criteria as a potential case either in the case-control study or incidence trend study, the local radiographers use the CR cassette reader to visualize the image and translate the image to PC-readable format and send it electronically to the trained panel of chest radiograph readers. We created a pool of eight primary chest radiograph image readers comprised of consultant pediatric radiologists and pediatric consultants who are based in Dhaka. They were trained and standardized to the WHO chest radiograph interpretation definitions for pediatric vaccine effectiveness studies by an international WHO-certified trainer for two days ( Table 7) ^21^,^22^. After this initial standardization process was completed the study pediatric pulmonologist (EDM) is facilitating ongoing, twice-annual re-standardization of the readers to the WHO protocol, in consultation with the WHO-certified trainer, for the duration of the project and also serves as the panel’s expert reader and provides quality control oversight of the panel.

**Table 7.** World Health Organization-defined pediatric antero-posterior chest radiograph findings used in the Bangladesh Pneumococcal Conjugate Vaccine Impact Assessment.

Quality	Interpretable	Image is interpretable for the presence or absence of endpoint consolidation or pleural effusion.
	Uninterpretable	Image is not interpretable for the presence or absence of endpoint consolidation or pleural effusion.
Classification	Endpoint Consolidation	A dense or confluent opacity that occupies a portion or whole of a lobe or the entire lung that may or may not contain air bronchograms.
	Other infiltrate (non-endpoint)	Linear and patchy opacities (interstitial infiltrate) in a lacy pattern, featuring peribronchial thickening and multiple areas of atelectasis; it also includes minor patchy infiltrates that are not of sufficient magnitude to constitute endpoint consolidation, and small areas of atelectasis that in children may be difficult to distinguish from consolidation.
	Pleural effusion	Presence of fluid in the lateral pleural space between the lung and chest wall that is spatially associated with a pulmonary parenchymal infiltrate (including other infiltrate) or has obliterated enough of the hemithorax to obscure any infiltrate; in most cases, this will be seen at the costo-phrenic angle or as a layer of fluid adjacent to the lateral chest wall; this does not include fluid seen in the horizontal or oblique fissures.
Conclusion	Primary endpoint pneumonia	Presence of endpoint consolidation or pleural effusion, as defined above.
	Other infiltrate	Presence of other (non-consolidation) infiltrates as defined above in the absence of a pleural effusion.
	No consolidation/infiltrate/effusion	Absence of consolidation, other infiltrates or pleural effusion.

Each digital image of a chest radiograph from a child with clinical pneumonia is independently read by two trained primary readers, selected randomly from the pool of eight readers. Readers are blinded to the clinical data and also the ultrasonographic images and interpretations. For interpretable radiographic images, if the interpretation by the two primary readers is identical for the presence or absence of WHO primary endpoint pneumonia, then the classification is considered final. If the classifications of the image by the two primary readers are discordant, the image is then sent to a third reader who is randomly selected from the remaining readers in the pool. The third reader is blinded to the initial classifications by the first two readers and the fact that they are serving as the third reader for that image. If the classification of the third reader agrees with one of the initial two readings, then the third reader interpretation is considered final. However, if the third reader does not agree with either of the first two readings, the image is sent to the study expert reader (EDM) and his reading is then considered final. In addition, a 20% random sampling of all the images are read by the expert reader (EDM) as a quality control measure and regular performance reports are generated as feedback to the radiograph reading panel. Initial management of the children is done based on clinical and bacteriological findings since radiological findings take 24–48 hours to be finalized. Once finalized, the image results are also shared with the treating physicians and may also be taken into consideration for further patient management given the initial treatment decision is made on the patient’s clinical presentation only. Individual reader performance is monitored throughout the study and refresher trainings and reader re-standardization to the WHO protocol are conducted every six months under the facilitation of the expert reader (EDM), in consultation with the WHO certified trainer. All images are archived at the site and catalogued by study ID and date. A written report on the chest radiograph result is provided to parents at the next routine household visit after the results become available or sooner if the child returns to the hospital clinic.

We initiated the incident chest radiograph case-control study two months after introduction of PCV10 in the national vaccine program in our study area. The age eligibility for the chest radiograph case-control study is shown in Table 8 and sample size requirement based on different PCV coverage scenarios is shown in Table 9. Cases are age-eligible children diagnosed with radiographically confirmed pneumonia at a study hospital. Control selection procedures are the same as the IPD case-control study.

**Table 8.** Age eligibility criteria for inclusion in radiographic and lung-ultrasound confirmed cases in case-control studies by study months.

Year	2015					2016											
	Aug	Sep	Oct	Nov	Dec	Jan	Feb	Mar	Apr	May	Jun	Jul	Aug	Sep	Oct	Nov	Dec
Month of case- control study	1	2	3	4	5	6	7	8	9	10	11	12	13	14	15	16	17
Minimum age of eligibility in completed months	3	3	3	3	3	3	3	3	3	3	3	3	3	3	3	3	3
Maximum age of eligibility	15	16	17	8	19	20	21	22	23	24	25	26	27	28	29	30	31
Year	2017																
	Jan	Feb	Mar	Apr	May	Jun	Jul	Aug	Sep	Oct	Nov	Dec					
Month of case- control study	18	19	20	21	22	23	24	25	26	27	28	29					
Minimum age of eligibility in completed months	3	3	3	3	3	3	3	3	3	3	3	3					
Maximum age of eligibility	32	33	34	35	35	35	35	35	35	35	35	35					

**Table 9.** Detectable odds ratio with 80% power and 0.05 type 1 error for radiographic and lung ultrasound studies.

Cases	Vaccine coverage	Matched controls per case	Detectable OR
1130	46%	1	0.766
1130	46%	2	0.800
1130	46%	3	0.813
1130	46%	4	0.820

***Data collection from cases and controls.*** An additional written informed consent is obtained from the parents of the selected case and control children. After consent is obtained, case and control households are visited to collect vaccination status data and other information that is required as covariates or potential confounders in the analysis, such as socio-economic status, as detailed for the IPD analysis.

### Objective 4: Lung ultrasound confirmed pneumonia case-control and incidence trend studies

In contrast to chest radiography, the use of lung ultrasound for the diagnosis of pneumonia is not yet considered standard of care or even part of medical curricula or guidelines from pediatric societies. A recent meta-analysis and several randomized clinical trials, however, suggest that lung ultrasound performs well as a diagnostic tool for the identification of pneumonia when compared to chest x-ray ^23^,^24^ and that outcomes are similar when if lung ultrasound were to replace chest x-rays ^25^. Recent studies by our research group conducted in Peru ^26^ and Nepal ^27^ suggest that lung ultrasound is a feasible and appropriate technology for the diagnosis of pediatric pneumonia in resource-poor settings. However, since these studies only used one or two study physicians, we were unable to assess the role of our standardized training program on the conduct and interpretation of lung ultrasound findings when using a large number of study physicians. Three portable ultrasound machines (Sonosite Edge, Bothell, WA) were placed at each of the three sub-district hospitals (Zakiganj, Kanaighat and Beanibazar) and used for lung ultrasound assessments. Study physicians underwent a standardized training course to learn the use of lung ultrasound in the diagnosis of pediatric pneumonia. As per the study protocol, the seven-day training course consisted of both theoretical and practical training. The first three days of training consisted of classroom learning of ultrasound basics and recognition of pathologies, and the next four-days were direct ultrasound training in a pediatric ward to properly identify lung ultrasound patterns in children consistent with pneumonia, other respiratory abnormalities or with normal findings ( Table 10). After completion of the seven-day training, the trainees undertook both a theoretical and practical competency assessment. All study physicians were required to achieve a passing grade of 80% to be considered standardized.

**Table 10.** Lung ultrasound findings used in the Bangladesh Pneumococcal Conjugate Vaccine Impact Assessment. Description of findings on lung ultrasound and definition of endpoint pneumonia, interstitial abnormality and atelectasis.

Quality	Interpretable	Ultrasound is interpretable for the presence or absence of endpoint consolidation, atelectasis, or interstitial abnormalities.
	Uninterpretable	Ultrasound quality is not interpretable for the presence or absence endpoint consolidation, atelectasis, or interstitial abnormalities. Ultrasound does not have all 24 clips recorded.
Classification	Endpoint Consolidation	Hypoechoic area or tissue pattern with loss or attenuation of distinct pleural lines.
	Air Bronchogram	Fluid or inflammation along the bronchial walls. This is visualized on ultrasound as punctate hyperechoic or hypoechoic images.
	B-Lines	Well defined hyperechoic comet-tail artifacts arising from the pleural line, spreading down, indefinitely erasing A lines and moving with lung sliding when lung sliding is present.
	Pleural Abnormality	Disruption along the pleural line that is not large enough to be measured as a consolidation.
	Shred Sign	Disruption of the pleural line, caused by consolidation or pleural effusion, that forces the pleural line to become discontinuous and move below the level of the consolidation.
	Pleural effusion	Presence of fluid in the lateral pleural space between the lung and chest wall. This is visualized on ultrasound as hypoechoic images in the pleural space.
	Primary Endpoint Pneumonia	Presence of consolidation that measures ≥ 1 cm or greater than 1 intercostal space, or a pleural effusion with any of the following; consolidation < 1cm, ≥ 3 B-lines, air bronchograms.
	Interstitial Abnormalities	Presence of artifacts consistent with ≥ 3 B lines or pleural abnormalities.
	Atelectasis	Presence of consolidation < 1 cm or smaller than 1 intercostal space.

In contrast to chest radiography where findings are consistent with anatomatical changes, findings on lung ultrasound are artifacts that are associated with anatomic changes and pathology and not with acute anatomic changes ( Table 10). We defined pneumonia on lung ultrasound as a as a consolidation ≥ 1 cm in size or greater than one intercostal space, or a pleural effusion with any of the following: consolidation < 1 cm in size, ≥ 3 B-lines or presence of air bronchograms. Interstitial abnormalities were defined as ≥ 3 B-lines or presence of pleural abnormalities. Atelectasis was defined as a consolidation < 1 cm in size or smaller than one intercostal space.

***Ultrasound procedures.*** The use of lung ultrasound began after all study pediatricians had at least one month of practice using the ultrasound machine for pneumonia diagnosis. At the onset of the study, all ultrasounds were conducted and interpreted by a study physician and re-assessed remotely by one of three expert physicians. However, as the volume of patients increased a two reader process was implemented with an expert reader only reviewing discrepancies. The study physician who conducted the ultrasound was the first reader. A second study physician randomly selected from the team would provide an independent second read. If there was disagreement in the interpretation of the lung ultrasound image between two physicians, then the image is reviewed by an expert sonographer who would then act as the ombudsman and provide a final diagnosis. We initially developed a standardized case-report form that was used by both the study physicians and expert readers. However, seven months into the study the case volume of child participants became too high, and we had to develop a shorter CRF that was easier to complete by the study physicians. The content of the CRF remained consistent throughout the course of the study, and the case report form for the expert readers remained the same.

 Procedures for the case-control and incidence monitoring studies are the same as those described under Objective 3. Follow-up visits for children with ultrasound-confirmed pneumonia may include a repeat ultrasound.

### Objective 5: Assessment on the impact on NP carriage

An assessment of the baseline rates of NP pneumococcal colonization among participants with radiograph-confirmed pneumonia began following the start of the chest radiograph study in September 2015. Physicians collect nasopharyngeal swabs on all consenting clinical pneumonia cases. Participants are requested to sign a separate consent for NP swab collection. NP specimens are collected using nylon flocked swabs in accordance with updated WHO core methods ^28^,^29^. A single NP specimen is collected from the posterior nasopharynx of each subject by inserting the swab, rotating it 180 degrees and removing it. The swab is inserted immediately into one mL of liquid STGG transport medium and transported on ice to the clinical laboratory in Sylhet. Swabs are frozen at -20°C and stored for up to seven days. All swabs of confirmed radiographic pneumonia cases are cultured for pneumococcus; the remaining swabs are discarded. In the lab, the specimen are vortexed, aliquoted into two separate specimens (one with the swab retained), and a sample inoculated into broth for enrichment. Following broth enrichment, the sample is plated onto a gentamicin-blood agar plate and incubated in a CO2 incubator for isolation of pneumococcus. An aliquot of the broth enrichment as well as the remaining STGG NP sample are frozen (−80°C). Pneumococci are detected based on morphology, optochin susceptibility and bile solubility.

To determine the density of pneumococcal colonization, a second sample from the STGG vial is subjected to 10-fold dilutions up to 10 . An amount of 100 μl of each dilution is plated onto gentamicin blood agar plates and the semiquantitative results are documented, based on the numbers on the plates with countable number of pneumococcal and dilution factors. Pneumococcal strains are serotyped by the capsular swelling procedure (quellung reaction) with type-specific anti-pneumococcal omni, pool, type or group, and factor sera (Statens Seruminstitute, Copenhagen, Denmark).

Data on PCV10 vaccination status are collected from parents of all participants enrolled in the NP carriage study at home visits if vaccination card is available. If the child’s parents fail to produce a card at enrollment or at their home but state a history of the child having received vaccines, the registry at the local immunization clinic registry is consulted. Demographic and socio-economic characteristics are captured once during the study on all participants via a household survey.

### Coverage survey and incidence trend analysis

 We have conducted two PCV coverage surveys, one in August-September 2016 and the other in August-September 2017. The surveys included simple random samples of 3,753 and 5,421 vaccine age eligible children stratified by age in months and union (with an average population of 25,000; there are 30 unions in the study area), respectively. This assures that every union’s coverage of <24 month-old children can be estimated to within ten percentage points (or less) with 95% confidence during any study month. The estimated vaccine coverage for the entire area for any given month will be more precise. These data will allow us to relate vaccine coverage to disease incidence via a Poisson regression model with one observation for each union-month.

### Training and quality control

***Community health workers.*** Before initiating data collection, CHWs are provided with training on surveillance activities, screening and identification of suspected IPD cases including pneumonia and meningitis in the community. The training includes theoretical discussions as well as real-life assessment of children in Sylhet Medical College Hospital. CHWs are also provided training on how to supervise VHWs. Field supervisors supervise and oversee CHW work at home. Supervisors meet with CHWs every two weeks and updates and continued training is provided. Supervisors accompany the CHWs twice a month to observe CHW activities and carry out independent home visit and data collection in a 5% random sample of households. Physicians conduct regular validation of CHW screening procedures and a refresher training is arranged when necessary.

***Physicians.*** All physicians receive standard integrated management of childhood illness (IMCI) training before starting screening and enrolment. Every 1–3 months throughout the project all study physicians receive intensive individual supervision of respiratory screening of children by the study pediatric pulmonologist (EDM), including immediate individual feedback as well as study reports in order to track individual study physician performance to identify those in need of remediation. In addition, study physicians receive twice annual refresher trainings facilitated by the study pediatric pulmonologist (EDM). Lumbar puncture is done by physicians who are trained on the technique at Dhaka Children Hospital or Sylhet Medical College Hospital. Physicians who carry out ultrasound are trained with a seven-day training course followed by on-site supervision and remote quality assurance by a pulmonologist with significant expertise in lung ultrasound for diagnosis of pediatric pneumonia.

A group of expert sonographers supervised the study physicians in performing the ultrasounds onsite for two weeks following the training course, and for a period of one month all ultrasound clips were transmitted to one of the two expert sonographers for confirmation of physician interpretations, prior to use of the study physician interpretations. We also conducted re-training and re-standardization throughout the study period, either as refreshers for current study physicians or to standardize new study physicians. We also had quarterly quizzes to review interpretation of lung ultrasound images. A total of 31 physicians underwent lung ultrasound training and standardization as part of this study. Moreover, to build local capacity in lung ultrasound, two study physicians were trained as local experts to conduct quality assessments and continued training. Separately, an expert sonographer over-read 20% of images for quality control purposes. All ultrasound clips were digitally and securely stored on a portable hard drive, and uploaded to a cloud server for interpretation (Ultralinq New York, NY).

### Data management, analysis plan and description of the nature of the variables to be derived

Data generated in the field reach the data management unit within three weeks and are entered in computers using a custom relational data entry system developed by our team (PCV v5.9). The system has built-in checks for range, consistency and validity of data to minimize errors. Data required for field monitoring and patient management (e.g., lab results) are entered in our field offices immediately after collection using an online web-based data entry system. Laboratory Management System Software is used to maintain Good Laboratory Practices. Most equipment is computerized. Data cleaning is performed periodically to ensure availability of cleaned data tables within five weeks of data collection. Data analysis is performed using STATA 13.

***Objective 1.*** Overall, vaccine type and non-vaccine type IPD rates will be estimated from the data generated in Objective 1 period.

***Objective 2.*** For descriptive purposes, the relative frequencies of the demographic, socioeconomic, matching, clinical, and other study variables will be compared for the cases and controls. We will conduct two separate case-control analyses, one using hospital controls and one using community controls. To adjust for multiple confounding variables as well as to evaluate effect modification, conditional logistic regression method designed for matched studies will be used ^30^. This analysis will provide adjusted odds ratios and its 95% confidence intervals (CIs). Since perfect age-matching may be difficult, age may remain a potential residual confounder because it is related to both disease and likelihood of vaccination. To handle this potential problem, vaccine efficacy (VE) estimates will be age adjusted by including a linear term for age in months in all models. Estimates of vaccine effectiveness will be calculated using the formula:

       VE = (1 − matched OR) × 100,

where OR indicates the odds ratio for receiving PCV in cases and controls ^31^. Case-control analyses for each group of controls (hospital controls and community controls) will be conducted separately and the results compared and discussed. Data from the surveillance component of the study will be analyzed to quantify the magnitude of the incidence of IPD and to determine the clinical and epidemiologic characteristics of IPD.

***Objective 3.*** Similar to IPD case-control study, we will conduct two separate case-control analyses for radiographically- confirmed pneumonia, one using hospital controls and one using community controls. To adjust for multiple confounding variables as well as to evaluate effect modification, the conditional logistic regression method designed for matched studies will be used in this analysis as well ^30^. Similar to the IPD analysis, we will also generate the adjusted OR and its 95% CI and since residual confounding by age may be an issue with this analysis as well, we will likely age adjust vaccine efficacy estimates by including a linear term for age in month in all models, as described for Objective 2. We will define VE as done in Objective 2, but in this analysis OR will indicate the odds ratio of radiologically confirmed pneumonia to PCV vaccination status ^31^. In the absence of any selection bias, the OR will estimate the incidence rate ratio.

The incidence rate of chest radiograph-confirmed pneumonia will be computed by dividing the number of radiograph-confirmed pneumonia cases by child years of observation. The 95% CI will be calculated using the Poisson distribution. Separate point and interval estimations for different types of endpoints as defined by the classifications of chest radiograph results will be computed.

To estimate the impact of the vaccine, the incidence rate ratio will be calculated by dividing the incidence rate in study months 13–24 by the incidence rate in study months 1–12; standard errors and 95% CIs will be reported. Poisson regression will be used to assess trends in the incidence rate over time. We will also investigate whether any clustering by study hospital or evaluating pediatrician is evident.

***Objective 4:*** Our analytic methodology for Objective 4 will be similar to that for Objectives 2 and 3, except that the endpoint will be ultrasound-confirmed pneumonia rather than radiographically- confirmed pneumonia.

***Objective 5:*** The primary study outcome will be prevalence of vaccine-type pneumococci (serotypes 1, 4, 5, 6B, 7F, 9V, 14, 18C, 19F, and 23F) among children with radiographically-confirmed pneumonia, stratified by vaccination status. We will calculate this prevalence rate for all children with X-ray confirmed pneumonia during a rolling calendar period, beginning the first week of NP swab collection. Standard deviations and 95% confidence intervals for the prevalence rates will be calculated. Rates will be calculated both unadjusted and adjusted for participant demographic characteristics. Rates will be plotted against time separately for vaccinated and unvaccinated participants using the midpoint of the interval as the time point shown on the x-axis, and with population prevalence of immunization superimposed, as in Loughlin *et al.* (2014) ^32^.

In a secondary analysis, we will use a multivariate logistic regression model to test the effect of time trend, vaccination status and the interaction of time and vaccination status on carriage of VT pneumococci in this population of pediatric Bangladeshi pneumonia patients. Other analyses may explore the joint effect of vaccination status and time on carriage of NVT pneumococci.

## Ethical approvals

The study protocol was approved by the Institutional Review Board (IRB) of the Johns Hopkins Bloomberg School of Public Health (IRB 00005421), and the Ethical Review Committee of the International Centre for Diarrhoeal Diseases Research, Bangladesh (PR-13095).

## Study status

At the time of writing this manuscript, fieldwork for all objectives has been completed with the exception of the IPD case-control and incident trend studies, which will be ongoing through June 2018. Data cleaning and preliminary analysis are currently underway.

## Dissemination of results

At this point, some preliminary data analysis has been completed. Five abstracts were submitted and accepted for presentation at the ISPPD-2018 conference in April 2018 in Melbourne, Australia. Data analysis will continue throughout 2018, and a dissemination seminar in Bangladesh will be held in late 2018 in collaboration with our main stakeholder, the Bangladesh Ministry of Health and Family Welfare. We anticipate that at least one dozen peer-reviewed publications will be produced from this study.

## Discussion

The GoB MOHFW introduced PCV10 on March 2015, making it the second South Asian country to add PCV to their national vaccine schedule. While developed countries have seen significant decreases in pneumococcal disease after the introduction of PCV vaccines, there are a number of challenges that should be considered when introducing a new vaccine in resource poor settings such as in Bangladesh ^33^,^37^. First, pneumococcus has over 90 different serotypes, and the currently licensed vaccines contain only either 10 or 13 of the different serotypes. Additionally, the serotype distribution of the pneumococci that cause disease varies by location, and some countries have observed serotype replacement ^38^,^40^. GAVI currently provides co-funding for the introduction of vaccines such as PCV. Countries such as Bangladesh will be required to bear the financial burden for the implementation of all the vaccines including PCV, which is very expensive. Because of these issues, the WHO recommends that all countries have an assessment of the introduction of PCV10 to determine its impact in each setting.

The Pneumococcal Conjugate Vaccine Impact Study, expected to be completed in mid-2018, is a prospective community- and facility-based observational cohort study with a variety of nested studies including case-control and pre-post studies to evaluate the impact of PCV10 in Bangladesh. We designed a comprehensive, multiple outcome impact assessment because each outcome has its own strengths and drawbacks. Classic impact assessments generally measure and compare rates of IPD in vaccinated and unvaccinated children. However, there are several limitations to such a design. For example, not all IPD is associated with bacteremia, and the majority of cases of pneumococcal pneumonia are blood culture negative ^41^. Therefore, estimating the impact of vaccine on IPD alone will underestimate the burden of pneumococcal disease and will underestimate the true value of the vaccine. Additionally, many children arrive at health facilities with recent or current antibiotic use, increasing the difficulty to isolate pneumococcus in the lab from their specimen ^42^. To account for the attenuated estimate expected when measuring IPD rates and to get a more complete assessment of the impact of PCV10 in our population, we have considered 3 additional outcomes: 1) chest radiograph-confirmed pneumonia; 2) lung ultrasound-confirmed pneumonia; and 3) NP carriage.

We have measured NP carriage because a precursor to disease is carriage of the bacteria in the nasopharynx. If the vaccine reduces VT NP carriage, then we would potentially expect the transmission of disease to be lower and that there will be less disease. However, while up to 70% of children may be colonized with the pneumococcus in the nasopharynx, a very small proportion of these children will become ill with this disease ^43^. Nevertheless, the reduction of NP carriage is one measure of the impact of the vaccine. Moreover, reduction of NP carriage also reduces the circulation of the organism in the community and thus confers protection to unimmunized children who live in the same community – “herd immunity” ^38^.

Although chest radiographs and lung ultrasound imaging outcomes are non-specific to pneumococcus, radiographically- and sonographically-confirmed pneumonia could be markers for bacterial pneumonia. Two placebo randomized controlled trials (RCTs) in The Gambia and South Africa evaluated VE of PCV against WHO-defined radiographic pneumonia in children ^44^,^45^. These studies validated the use of the WHO-defined radiographic pneumonia definition in PCV impact studies by observing a VE of 20% (95% confidence interval (CI), 2%, 35%) in South African children without HIV-infection, and a VE of 37% (95% CI, 25%, 48%) among Gambian children against this endpoint. These RCT findings support our decision to include the WHO-defined radiographic pneumonia endpoint (i.e., primary endpoint pneumonia) in this study.

Given PCV VE is now well established against radiographic pneumonia in settings outside of South Asia, a placebo RCT designed study is no longer ethical. We are therefore utilizing both case-control and time-series designs in this PCV impact study to assess VE against radiographic pneumonia. One recent case-control study from The Gambia reported mixed results using WHO-defined radiographic pneumonia among children 3–59 months old ^46^. In this study the authors used conditional logistic regression, as we also plan to do, but found that they were overall underpowered to show VE, largely due to the high percentage of controls receiving PCV. Their best estimate for VE was an adjusted OR of 0.57 (95% CI, 0.30, 1.08) among children 3–11 months old. One important strength of this study from The Gambia is that the authors also conducted a time series analysis on the same dataset and found a statistically significant reduction in the incidence of WHO-defined radiographic pneumonia across all pediatric age ranges in children below five years of age. Utilizing multiple analytic strategies allowed the authors to better estimate the impact of PCV.

Another recent case-control study from South Africa did find statistically significant VE among vaccine eligible children utilizing a modified WHO-defined radiographic pneumonia definition that included children as cases if they were found to have the WHO radiographic classification of ‘other infiltrate’ along with an elevated C-reactive protein inflammatory biomarker >40 mg/L ^47^. WHO-defined radiographic ‘other infiltrate,’ however, has been shown to be a notoriously unreliable imaging endpoint as multiple studies have reported low published kappa levels between image readers ^21^,^48^. Most recently, the Pneumonia Etiology Research for Child Health study unsuccessfully attempted to standardize and adjudicate imaging readers to this ‘other infiltrate’ endpoint, finding a Cohen’s kappa of only 0.15 between the first two radiographic readers ^48^. The authors concluded that despite a rigorous standardization process imaging reader agreement remained poor for the classification of WHO ‘other infiltrate.’ Based on this literature, we will use WHO-defined radiographic pneumonia as our radiographic case definition and will not consider ‘other infiltrate.’ Instead, we plan to balance the potential limitation of being insufficiently powered to detect VE of radiographic pneumonia using the case-control design by conducting a complementary time series analysis using this same imaging endpoint.

Including both chest radiograph and lung ultrasound imaging, compared to IPD only, will likely provide a better understanding of true disease burden estimates and impact of the vaccine ^49^. While the chest radiograph is a useful tool to diagnose pneumonia the machines are not easily mobile, expensive, expose children to ionizing radiation, and they provide only a one-dimensional image for assessment. Comparatively, the lung ultrasound method of diagnosis is potentially easier to perform, can be done at the patient’s bedside, and it produces video images of multiple locations of the chest without ionizing radiation exposure, permitting the reader additional perspectives of the child’s lungs for interpretation. Additionally, physicians can be trained to use the lung ultrasound machines and diagnose the patient, and treatment does not need to be delayed while waiting for a radiologist to read radiographic images. Using lung ultrasound for diagnosing pneumonia, however, is still in investigational stages, and its efficacy for measuring pneumonia is still to be determined ^50^,^51^.

## Data availability

No data is associated with this article.
