# Supplementary material for: Pneumococcal Conjugate Vaccine impact assessment in Bangladesh
Source: Gates Open Res. 2018 Apr 26;2:21. [Version 1] doi: 10.12688/gatesopenres.12805.1 (PMC6030398; doi:10.12688/gatesopenres.12805.1)
Supplement: Peer review history

# Peer review: Referee response for version 1

**DOI:** 10.21956/gatesopenres.13869.r26536

**Published:** 2018-07-02

**Authors:** Marie R. Griffin

**Affiliations:** 1 Department of Health Policy, Vanderbilt University Medical Center, Nashville, TN, USA

This is a very ambitious study of impact of PCV10 on serious morbidity in children which should yield important information. The case control methodology is appropriate when pneumococcal serotype can be ascertained. However, I agree with Dr. Dagan that performing a case control study for all cause pneumonia may underestimate vaccine impact. For the ecologic analyses of impact, it would be good to have some assurance that surveillance was similar and as complete as possible, before and after vaccine introduction. If uptake of vaccine was sufficiently high over a short period of time, then use of a time series analysis rather than strict before – after may strengthen causal inference. Other analytic techniques could include difference in difference approaches; that is, before-after in regions with high vs low vaccine uptake.

 I applaud incorporating ultrasound into this study and wonder if it might also be the appropriate technology for resource rich as well as resource poor settings.

 Given that the NP carriage data (objective 5) will only be of pneumonia cases, and not of the source population, it is not clear how these data should be interpreted. This is different from the study cited, which looks at carriage in a population sample.

 It is unclear from the protocol if any changes that might influence detection of cases has occurred over the study period. Some background on antibiotic use in this population would provide context. For example, how available are antibiotics and how frequently administered outside traditional health care system. It would seem important to document whether there were changes in antibiotic use due to either vaccine effectiveness or to the educational efforts that accompanied this vaccine intervention and evaluation. It would be important to document that efforts to provide a definitive diagnosis of pneumonia would also result in timely antibiotic administration for children with danger signs.

I have read this submission. I believe that I have an appropriate level of expertise to confirm that it is of an acceptable scientific standard, however I have significant reservations, as outlined above.

# Peer review: Referee response for version 1

**DOI:** 10.21956/gatesopenres.13869.r26535

**Published:** 2018-07-02

**Authors:** Ron Dagan

**Affiliations:** 1 The Faculty of Health Sciences, Ben-Gurion University of the Negev, Beer-Sheva, Israel

This represents an ambitious, generally well-thought and well-designed study. There are some difficulties in obtaining comprehensive data and high quality data for the population studied, but I believe this is addressed by the investigators. The very ambitious approach to pneumonia impact is to be commended. We definitely need prospective studies on the topic of PCV impact on respiratory infections. I have a few minor points to raise, and one major point as remarks. I believe that the major point (herebelow) in regard to the analysis of pneumonia should be taken seriously to prevent mistakes previously made by other investigators.

1. Page 6, 1 paragraph, right column, line 1-2: Clinical pneumonia is not considered usually as IPD, since in most cases it is a result of aspiration. It is important not to analyze this as IPD unless *S. pneumoniae* was isolated from blood or pleural effusion.
2. Table 2: Why exclusion criteria for obtaining blood or CSF culture? Previous enrollment or previous recent antibiotics will not always prevent **positive cultures in many circumstances.
3. Pneumonia: In general, I commend the X-ray approach for the Dg of consolidated pneumonia. I also am happy with the intention to test for incidence dynamics as this represent real-life impact of PCV10. However, case-control studies for pneumonia (excluding pneumonia with positive pneumococcal culture and serotypes) are inappropriate. Although, case-control studies were conducted by several investigators, the method is inappropriate for the following reasons:
  a. Consolidated pneumonia is often caused by *S. pneumoniae* albeit not always.
  b. Even if caused by *S. pneumoniae*, it can be caused by non-vaccine serotypes *S. pneumoniae*.
  c. Introduction of PCV will deplete cases of consolidated pneumonia for caused by vaccine-serotypes, if the vaccine is efficacious.
  d. The more the vaccine is efficacious, and the more the vaccination program achieves high vaccination coverage, the less likely will the remaining consolidated pneumonia be seen covered by vaccine serotypes.
  e. Therefore, in other words, the higher the impact of PCVs, the less likely is the remaining consolidated pneumonia to be caused by vaccine serotype. Therefore, the more the vaccine impacts on pneumonia, the less the vaccine is effective against the remaining pneumonia.
  f. Thus, immediately after the vaccine introduction, case-controls studies for effectiveness of the vaccine, when the pathogen cannot be identified will show lower-than-real effectiveness, and the trend will be more severe as more children are vaccinated.
  g. Of course, this is different when cases for whom vaccine serotype *S. pneumoniae *is isolated, since we only count those that are not supposed to be decreased by PCVs.
  I therefore strongly suggest not to conduct case-control studies for symptomatic pneumonia, but rather make all efforts to study incidence dynamics, thus determine ***impact*** rather than ***effectiveness***.
4. Similar remarks are for ultrasound confirmed pneumonia cases

I have read this submission. I believe that I have an appropriate level of expertise to confirm that it is of an acceptable scientific standard, however I have significant reservations, as outlined above.

# Peer review: Referee response for version 1

**DOI:** 10.21956/gatesopenres.13869.r26383

**Published:** 2018-06-25

**Authors:** Peter Waiswa

**Affiliations:** 1 Maternal, Newborn and Child Health Centre of Excellence, School of Public Health, College of Health Sciences, Makerere University, Kampala, Uganda; 2 IHCAR, Division of Global Health, Karolinska Institutet, Stockholm, Sweden

Thanks for the opportunity for me to review this important protocol. The protocol is well written. However, the main challenge is that of assessing the vaccine impact especially surveillance by using CHWs and the fact that there are multiple providers some information can be a challenge to collect with precision. I know these CHWs and providers are being engaged through training and supervision, but ensuring that all incident cases of disease are captured can be a daunting task. In addition, I find the ultrasound part of the study to be just an opportunistic one - not necessarily part of the PCV impact assessment but maybe it is good as a bonus  in the study. Finally, the authors are publishing this protocol almost at the end of the study - can they discuss a little more of what they had initially planned/designed and what changes they have so far made and why?

I have read this submission. I believe that I have an appropriate level of expertise to confirm that it is of an acceptable scientific standard.
